# Coevolution of hytrosaviruses and host immune responses

**DOI:** 10.1186/s12866-018-1296-3

**Published:** 2018-11-23

**Authors:** Henry M. Kariithi, Drion G. Boucias, Edwin K. Murungi, Irene K. Meki, Güler Demirbaş-Uzel, Monique M. van Oers, Marc J. B. Vreysen, Adly M. M. Abd-Alla, Just M. Vlak

**Affiliations:** 1 Biotechnology Research Institute, Kenya Agricultural and Livestock Research Organization, P.O Box 57811, Kaptagat Rd, Loresho, Nairobi, 00200 Kenya; 2 Insect Pest Control Laboratory, Joint FAO/IAEA Division of Nuclear Techniques in Food and Agriculture, Wagrammer Straße 5, A-1400 Vienna, Austria; 3 Entomology and Nematology Department, University of Florida, 970 Natural Area Drive, Gainesville, FL 32611 USA; 4 Department of Biochemistry and Molecular Biology, Egerton University, P.O. Box 536, Njoro, 20115 Kenya; 5 Laboratory of Virology, Wageningen University and Research, 6708 PB Wageningen, The Netherlands; 6 Present Address: US National Poultry Research Centre, Southeast Poultry Research Laboratory, USDA-ARS, 934 College Station Road, Athens, GA 30605 USA

**Keywords:** *Hytrosaviridae*, *Musca domestica*, *Glossina* spp., Symbionts, *Muscavirus*, *Glossinavirus*, Apoptosis, RNAi, miRNA

## Abstract

**Background:**

Hytrosaviruses (SGHVs; *Hytrosaviridae* family) are double-stranded DNA (dsDNA) viruses that cause salivary gland hypertrophy (SGH) syndrome in flies. Two structurally and functionally distinct SGHVs are recognized; *Glossina pallidipes* SGHV (GpSGHV) and *Musca domestica* SGHV (MdSGHV), that infect the hematophagous tsetse fly and the filth-feeding housefly, respectively. Genome sizes and gene contents of GpSGHV (~ 190 kb; 160–174 genes) and MdSGHV (~ 124 kb; 108 genes) may reflect an evolution with the SGHV-hosts resulting in differences in pathobiology. Whereas GpSGHV can switch from asymptomatic to symptomatic infections in response to certain unknown cues, MdSGHV solely infects symptomatically. Overt SGH characterizes the symptomatic infections of SGHVs, but whereas MdSGHV induces both nuclear and cellular hypertrophy (enlarged non-replicative cells), GpSGHV induces cellular hyperplasia (enlarged replicative cells). Compared to GpSGHV’s specificity to *Glossina* species, MdSGHV infects other sympatric muscids. The MdSGHV-induced total shutdown of oogenesis inhibits its vertical transmission, while the GpSGHV’s asymptomatic and symptomatic infections promote vertical and horizontal transmission, respectively. This paper reviews the coevolution of the SGHVs and their hosts (housefly and tsetse fly) based on phylogenetic relatedness of immune gene orthologs/paralogs and compares this with other virus-insect models.

**Results:**

Whereas MdSGHV is not vertically transmitted, GpSGHV is both vertically and horizontally transmitted, and the balance between the two transmission modes may significantly influence the pathogenesis of tsetse virus. The presence and absence of bacterial symbionts (*Wigglesworthia* and *Sodalis*) in tsetse and *Wolbachia* in the housefly, respectively, potentially contributes to the development of SGH symptoms. Unlike MdSGHV, GpSGHV contains not only host-derived proteins, but also appears to have evolutionarily recruited cellular genes from ancestral host(s) into its genome, which, although may be nonessential for viral replication, potentially contribute to the evasion of host’s immune responses. Whereas MdSGHV has evolved strategies to counteract both the housefly’s RNAi and apoptotic responses, the housefly has expanded its repertoire of immune effector, modulator and melanization genes compared to the tsetse fly.

**Conclusions:**

The ecologies and life-histories of the housefly and tsetse fly may significantly influence coevolution of MdSGHV and GpSGHV with their hosts. Although there are still many unanswered questions regarding the pathogenesis of SGHVs, and the extent to which microbiota influence expression of overt SGH symptoms, SGHVs are attractive ‘explorers’ to elucidate the immune responses of their hosts, and the transmission modes of other large DNA viruses.

## Background

The salivary gland hypertrophy viruses (SGHVs) belong to the *Hytrosaviridae* [1], a relatively new family of insect double-stranded DNA (dsDNA) viruses that infect cyclorrhaphan flies with distinct ecologies and evolutionary histories. Known SGHV hosts are the hematophagous *Glossina* species (tsetse fly), filth-feeding *Musca domestica* Linnaeus (common housefly), and most probably the phytophagous *Merodon equestris* Fabricius (bumblebee-mimic fly) [2]. In the host, SGHV infection and replication results in the swelling of the salivary glands (SGs) and thereby producing diagnostic SG hypertrophy (SGH) syndrome [3]. SGVHs are enveloped and rod-shaped viruses that contain a single circular dsDNA genome [4–6]. Structurally, SGHVs are reminiscent of the well-studied baculoviruses [7] with which they phylogenetically form a monophyletic group together with other nuclear-replicating large dsDNA viruses such as the nudiviruses and nimaviruses [8]. Functionally however, SGHVs are readily distinguished from baculoviruses, in view of the absence of occlusion bodies, and, contrary to baculoviruses, SGHVs rarely kill their host (i.e. lower lethality) [9].

The SGHVs primarily replicate in adult flies, and cause a chronic infection that leads to reproductive dysfunctions [10]. In certain cases, such as in the tsetse fly mass-production facilities, GpSGHV can switch from asymptomatic to symptomatic infections, eliciting epizootics that decrease the flies’ productivity, which can ultimately lead to the collapse of the colony [11]. Significantly, the SGHVs replicate in select host gland tissues, but no viral replication has been observed in cell lines established from homologous or heterologous insect hosts [12]. The lack of SGHV-susceptible cell/tissue cultures has hindered detailed genetic studies of SGHVs. Phylogenetically, the GpSGHV 190 kb genome has limited gene (open reading frame; ORF) homology to the MdSGHV 124 kb genome [4–6], and this formed the basis for placing these viruses in two separate genera within the family *Hytrosaviridae* (*Glossinavirus* and *Muscavirus*, respectively). Neither of these viruses could be placed within any of the other established dsDNA virus families [7, 13].

The sole member of the genus *Muscavirus*, the *M. domestica* SGHV (MdSGHV), infects and causes only symptomatic SGH in houseflies [14, 15]. Topical exposure or injection of MdSGHV into adult houseflies results in overt SGH and total shutdown of oogenesis, thus inhibiting any potential for the vertical transmission of this virus [15, 16]. MdSGHV is globally distributed within populations of the synanthropic housefly [17], a highly mobile insect that moves several kilometers in search of feeding and oviposition sites associated with livestock keeping [18]. Sequence analysis of selected genes from different MdSGHV isolates revealed low polymorphisms between the isolates [17]. This low viral polymorphism potentially reflects the close associations of the highly gregarious domestic housefly with human movements/migrations, which may in turn influence the frequency of MdSGHV-housefly interactions. Within housefly populations, MdSGHV induces variable rates of SGH prevalence (e.g. 0–40% in North Florida dairy farms [19, 20]), potentially related to the fly’s seasonal densities at the various sampling sites. Since natural selection favors transmission modes that provide pathogens with the greatest fitness gains [21], the highly transient housefly densities may have evolutionarily favored horizontal transmission for MdSGHV.

The *Glossinavirus* type species, *G. pallidipes* SGHV (GpSGHV) exists predominantly in an asymptomatic infection state in tsetse flies, but certain unknown (biotic and abiotic) factors can trigger development of overt SGH symptoms in apparently healthy individuals [2, 22]. Intra-hemocoelic injection of GpSGHV does not induce overt SGH symptoms in the parental generation, rather, the symptoms develop in some of the F_1_ progenies [23]. Contrary to the globally distributed MdSGHV, GpSGHV is specific to *Glossina* species, which are restricted to sub-Saharan Africa. Tsetse fly distribution is principally determined by habitat, environmental conditions and host animal dynamics. Compared to the highly mobile houseflies, tsetse flies are fairly sedentary, and make random movements of only 150–550 m per day [24]. Based on selected viral genes, GpSGHV has a lower polymorphism than MdSGHV [17, 25]. Of all the *Glossina* species (in both field and wild conditions), it is only *G. pallidipes* that often exhibits overt SGH symptoms. Given that an asymptomatic (persistent) infection signifies the best adapted or most successful virus-host coevolution [26], *G. pallidipes* is arguably the most recent GpSGHV host, which partially explains the absence of overt SGH symptoms in other *Glossina* species [27].

This review evaluates the coevolution of SGHVs [4–6] and their hosts [28, 29]. Phylogenetic relatedness is determined for the immune genes in *G. pallidipes* and *M. domestic* in relation to orthologs/paralogs from the model insect, *Drosophila melanogaster*, and the African malaria mosquito, *Anopheles gambiae*. Inferences are made from other virus-host systems for which the immune genes have been identified and characterized.

## Host-range specificity, transmission dynamics and pathogenesis of SGHVs

### Infection dynamics of MdSGHV in houseflies

Examination of MdSGHV-infected houseflies revealed that SGH symptoms were due to the hypertrophy of the nucleus (nucleocapsid assembly) and cytoplasm (viral membrane acquisition) of the SG cells [30]. In addition to replication in the SGs, MdSGHV undergoes limited replication in the housefly’s corpora-allata/cardiaca (CA/CC) glands, the sites that produce neurohormones and juvenile hormones that regulate reproduction and metamorphosis [16]. Reverse transcriptase quantitative PCR (RT-qPCR) analysis demonstrated that replication of MdSGHV in non-glandular tissues blocked the transcription of hexamerin and yolk proteins [15].

Maintaining uninfected houseflies together with SGH-positive flies at various densities in cages with a shared food source for 10 filial generations resulted in low percentages (~ 10%) of SGH-positive houseflies that persist throughout the generations (~ 12 weeks) [14]. Significantly, unlike the GpSGHV*-Glossina* system, these MdSGHV infections did not cause the collapse of the housefly populations. This virus is presumably transmitted *per os* amongst houseflies that feed on shared food substrates [19], but this transmission mode is apparently insufficient to sustain a high prevalence in housefly populations. In the laboratory, force-feeding newly-eclosed (<2 h-old) flies with MdSGHV suspensions induced overt SGH symptoms in ~ 53% of the individuals [17]. In nature however, such newly eclosed flies do not imbibe food until after several hours, which, within 12–24 h post-eclosion allows synthesis of the peritrophic matrix (PM) that protects the gut and allow the flies to become highly resistant to orally-ingested virus [17, 20]. During feeding, an infected housefly regurgitates digestive fluids (including salivary secretions) onto the food substrates thereby releasing copious amounts of infectious viral particles [31]. Combined with the fact that topical virus application to older flies (resistant to *per os* challenge) induces overt SGH symptoms [32], these results suggests that MdSGHV is exposed to and transmitted to healthy conspecifics through the cuticle wounds when flies feed en masse at virus-contaminated sites [33]. Moreover, laboratory-produced houseflies contract MdSGHV infections when introduced into virus-contaminated fly cages [19].

The absence of both asymptomatic infection state, and lack of either vertical or sexual transmission of MdSGHV in house flies raise the question of how MdSGHV has evolved to ensure its persistence within natural fly populations, particularly during low seasonal fly densities. This question is further compounded by the absence of occlusion bodies in SGHVs, which in the case of baculoviruses facilitate long-term survival of baculoviruses outside of their hosts. Potentially, the insect saliva in combination with released contents of the insects’ crop organ stabilizes the MdSGHV particles released during the feeding events. Cage studies have shown that the MdSGHV maintains a low frequency of infection over multiple fly generations [14]. In nature, the gregarious nature and high densities of houseflies in animal farms provide an avenue for increased exposure of flies to MdSGHV infections (via cuticle); such an exposure is a requisite for the maintenance of this virus in the fly populations. Alternatively, MdSGHV may reside asymptomatically in reservoir hosts, providing an additional avenue for virus maintenance. For example, under laboratory conditions, other muscids such as the obligate hematophagous stable fly (*Stomoxys calcitrans*), a larval predator of the housefly, and the black dump fly (*Hydrotaea aenescens*), support limited viral replication but without concomitant expression of overt SGH symptoms [32, 34]. Whether these or other muscids that occur sympatrically with the housefly can transmit infectious MdSGHV particles to healthy houseflies remains to be tested. It should be noted that tsetse fly species exhibit resistance to MdSGHV injections.

### Infection dynamics of GpSGHV in tsetse flies

Different *Glossina* species show wide variations in their susceptibilities to GpSGHV infection. Using six *Glossina* spp. derived from laboratory colonies, Demirbas-Uzel et al. [35] recently showed that *G. pallidipes* and *G. brevipalpis* were the most susceptible and refractory to the virus, respectively. Meki et al. [27], observed that 15% of wild-caught *G. brevipalpis* had asymptomatic GpSGHV infections as compared with 0–100% of asymptomatic infections in different *G. pallidipes* populations. Moreover, whereas only one GpSGHV haplotype infected *G. brevipalpis* flies, *G. pallidipes* harbored 13 different haplotypes [27]. Hypothetically, GpSGHV can be horizontally transmitted in the wild when cohorts of flies feed on the same wild animal [27], but it is unclear whether different tsetse species in the wild populations feed together on the same animal. In the normal type of feeding on an animal host, tsetse flies typically exhibit ‘pool-feeding’ whereby they lacerate blood capillaries, resulting in hemorrhage, which is rapidly imbibed [24]. After the flies cease feeding, small blood pools form around the capillaries, which if the deposited saliva contains infectious viral particles, other flies may become infected, thus aiding in virus dissemination within fly populations. In comparison, a non-systemic virus transmission mode through co-feeding on aviremic, non-clinical vertebrate hosts has been demonstrated for the vesicular stomatis virus in the striped black fly, *Simulium vittatum* [36]. However, most examined tsetse species display asymptomatic infections, and the number of virus particles deposited during feeding is much lower (~ 10^2^ viral genome copies) as compared with the levels deposited by symptomatic flies (~ 10^6^ genome copies) [22]. In any case, the dynamics of GpSGHV transmission probably depend on the feeding behavior of individual *Glossina* species, their feeding preferences, feeding time, proximity (in terms of time and space) of uninfected flies to infected flies, and the susceptibility to virus infection within the particular fly populations. On this note, it is known that for some tsetse species such as *G. pallidipes* living in the same habitat, each animal host (e.g. bovids) can support feeding of more than 1000 flies, with each host giving almost 300 blood meals daily [37].

The long-lived (120–150 days) solitary tsetse fly has a life history distinct from the short-lived (15–30 days) gregarious housefly, which may influence virus transmission. Tsetse flies reproduce by adenotrophic viviparity and females retain fertilized eggs, and feed larvae with intrauterine “milk” produced by the milk glands, which are highly modified accessory glands [38]. Females give birth to fully mature third-instar larvae, that upon larviposition burrow into soil and pupate within few hours [39, 40]. Under laboratory conditions, GpSGHV is transmitted horizontally and vertically [22], and in *G. pallidipes* females, the virus undergoes limited replication in the milk gland cells providing a conduit for its ingress into developing larvae/pupae [23]. Adults of the F_1_ *G. pallidipes* produced by virus-injected mothers display high prevalence of SGH and reproductive dysfunctions.

Recent data have indicated that when GpSGHV is injected into the third-instar larvae of *G. pallidipes*, the adults that develop from these larvae and their subsequent F_1_ progenies release negligible amounts of infectious viral particles via saliva during feeding [41]. These observations suggest that the balance between vertical and horizontal transmission in general is associated with significant changes in virulence of several pathogens [21]. For example, promotion of vertical over horizontal transmissions made bacteriophages less virulent [42]. Another example is the selection for reduced pathogenesis observed in the cucumber mosaic virus, after vertical serial passages in *Arabidopsis thaliana*, but not following horizontal passages [43]. Similarly, when subjected to long-term vertical transmission in its *Paramecium* host (>800 host generations), the parasitic bacterium *Holospora undulata* lost infectivity when shifted to horizontal transmission [44].

Within laboratory-bred and wild tsetse populations, GpSGHV typically causes asymptomatic infections [25]. There is much debate whether the GpSGHV asymptomatic infection represents a sublethal, persistent infection state, or it is a truly latent state. During persistent infections, a virus remains in specific cells of infected individuals, and is accompanied by a perpetual low-level production of virions, but without excessive cellular damages [45]. During a latent infection, viral genomes and proteins are present in infected cells for a certain period, but without demonstrable formation of infectious viral particles [26]. Notably, a virus can cause both persistent and latent infections in the same host at the same time, but in different cells or tissues, which may or may not be dictated by the tissue tropism of the virus [26, 45]. It is speculated that during asymptomatic infections, GpSGHV exists in both persistent and latent infection states at the same time. For instance, the release of low amounts of virus (~ 10^2^ viral genome copies) via saliva during feeding by an asymptomatic fly [22] supports GpSGHV persistent infection state in the SG cells, i.e. the virus replicates at such low levels that small amounts of viral particles are produced in the SGs without development of overt SGH symptoms. At the same time, the virus may latently infect non-salivary gland tissues in which viral DNA is detectable but no transcripts, for instance in the tracheal cells [3]. In both cases, the virus does not induce SGH symptoms or reproductive dysfunctions [22].

It is unlikely that SGHVs integrate into host genomes as proviruses, since using GpSGHV genes as probes did not indicate such integration. Maintenance of asymptomatic tsetse flies at high densities in fly holding cages and feeding the flies using the in vitro membrane feeding technique, as applied in mass-rearing facilities and some laboratories, increases opportunities for flies to imbibe infectious viral particles released via saliva [46]. Over time, the increased viral titers induce SGH that results in release of increased virus particles leading to additional symptomatic infections culminating in SGH epizootics that can destroy entire fly colonies [2, 6, 11]. Laboratory assays have demonstrated that GpSGHV delivered orally or by injections into asymptomatic (or uninfected) females increased virus titers, but did not induce SGH symptoms or reproductive dysfunctions [23, 35]. Examination of the SGs from symptomatic F_1_ progeny revealed that unlike the MdSGHV that induces both nuclear and cellular hypertrophy (enlarged cells incapable of replication), GpSGHV induces cellular hyperplasia (enlarged cells capable of replication) resulting in the observed SGH symptoms [2, 16].

Compared to MdSGHV, the asymptomatic (or persistent) infection is potentially advantageous to GpSGHV by ensuring transmission and maintenance of progeny virus over a long period of time (fly-to-progeny) [23]. The asymptomatic infection could facilitate multiple infections by lineages of the same virus or different viruses, thus leading to viral complexity and evolution of novel virus haplotypes because these may confer to the virus competitive fitness advantage to survive in the host. Lastly, by not inducing disease in the asymptomatically infected individuals, the virus can be transmitted horizontally (intra- and inter-species) via salivary secretions to other flies, particularly under laboratory settings where several tsetse fly species feed on the same feeding membrane, and thus maintained within the fly populations. In view of these advantages, one could conclude that GpSGHV has evolutionarily selected asymptomatic infection as a survival strategy.

## The reciprocal tripartite SGHV-host-symbiont interactions

### Symbiont-mediated ‘priming’ of the host immune system

Insects with restricted diets such as the hematophagous tsetse flies and the plant sap-feeding aphids, are furnished with specialized mycetomes containing symbionts that synthesize essential nutrients or digest and detoxify ingested foods. Tsetse flies harbor, depending on the species, a unique community of three bacterial symbionts, i.e. *Wigglesworthia*, *Soldalis,* and *Wolbachia* [47]. Similar to GpSGHV, the three symbionts are maternally transmitted transovarially or via the milk gland secretions to the developing larva [23, 47, 48]. Houseflies on the other hand, appear to be either devoid or possess low densities of the symbionts found in the tsetse flies. Bahrndorff et al. [49] detected *Wolbachia* infections in less than 4% of *M. domestica* females collected from 10 widely distributed farms in Denmark. Perhaps the low *Wolbachia* densities are transiently associated with the housefly, and are without any perceptible functional significance.

The presence and densities of these symbionts can influence the immune competence of the host [50]. For example, *Wolbachia* upregulates at various levels the transcription of immune genes upstream of several pathways (e.g. Imd, Toll, JNK, RNAi, JAK/STAT, autophagy, phagocytosis, melanization, etc.) in mosquitoes, *Drosophila*, silkworms, and some parasitoid wasps [51, 52]. *Wolbachia* also mediates strain-dependent protection of *Drosophila* against infections with the *Drosophila* C virus (DCV), flock house virus (FHV) and cricket paralysis virus (CrPV) [53, 54] as well as some mosquito viruses [55]. *Wolbachia* is presumed to facilitate its own persistence and maintain its close association with the host insect by modulating the immune responses of the insect hosts [56]. The *Wolbachia*-modulated immune effectors include cecropins, defensins, thioester-containing proteins (TEPs), C-type lectins (CTLs), reactive oxygen species (ROS), relish 1 (REL1), Spätzle 1A (Spz1A), and attacins [57, 58]. Stable introduction of some *Drosophila*-derived *Wolbachia* strains into *Aedes aegypti* primed immune effector genes in the transinfected mosquito, and consequently increased *Wolbachia* replication [59]. The increased *Wolbachia* titers interfered with pathogenesis and/or dissemination of dengue virus (DENV) in the transinfected *Ae. aegypti*. In another study, resident *Wolbachia* improved refractoriness of *Culex quinquefasciatus* and *D. melanogaster* to West Nile virus (WNV) infections by significantly reducing the viral titers and transmission during feeding compared to their *Wolbachia*-free counterparts [60]. However, *Wolbachia* can also shift the balance towards the virus in virus-host interactions. For instance, *Wolbachia* enhanced the susceptibility of the African armyworm *Spodoptera exempta* to infection by *S. exempta* nucleopolyhedrovirus (SpexNPV) and increased SpexNPV-induced mortality [61]. Is should however be noted that in addition or alternative to direct priming of the insect’s immunity, *Wolbachia* could also indirectly impact on the viral titers in the host. For example, *Wolbachia* could reduce viral replication by competing for cellular space and resources [62], a scenario which could assist the host’s immune system to suppress replication of remnant progeny viruses. Alternatively, *Wolbachia* could affect the vesicular transport apparatus within the infected cell that are required for viral trafficking within the infected cell cytoplasm, or produce certain molecules (e.g. via its type IV secretion system) that may directly impede the virus [63]. This is particularly due to the overlapping tissue tropism between the virus and the symbiont, and the potentially strong natural selection pressure for the two organisms to increase their chances for maternal transmission [23, 64].

*Wigglesworthia* does not directly confer pathogen resistance to its tsetse host, but is required for maturation during larval development and for proper functioning of the immune system in adult tsetse flies [50, 65]. The role of *Sodalis* in tsetse is largely unclear, but certain *Sodalis* genotypes are postulated to enhance both the tsetse’s susceptibility to trypanosome infections and its innate vectorial competency for transmission of the parasites [66] via a complex of biochemical mechanisms. Moreover, *Sodalis* produces many enzymes that impact various host metabolic and biosynthetic processes such as nutrient uptake, cellular transport, protein folding, and redox metabolism [67]. One could therefore logically infer that, *Sodalis* indirectly influences the outcomes of virus infection.

### Correlation between symbiont densities and SGH prevalence

The absence (or low densities) of *Wolbachia* is thought to contribute to the expression of SGH symptoms in laboratory-bred *G. pallidipes* colonies as compared with other *Glossina* species that usually do not show overt SGH symptoms [23]. In protective *Wolbachia*-host combinations, high densities of the *Wolbachia*, and the frequency with which this symbiont is maternally inherited are a prerequisite [68, 69]. Although up to 100% of tsetse flies in laboratory colonies can be *Wolbachia*-infected, the prevalence of infection significantly varies amongst wild tsetse populations. Doudoumis et al. [70] observed that *Wolbachia* prevalence varied among tsetse species, i.e. 100% and 90–100% in laboratory and wild *G. m. morsitans*, respectively, 100% in laboratory *G. m. centralis*, 52–100% in wild *G. austeni*, 2–41% in wild *G. brevipalpis*, 0.3% and 0–8.5% in laboratory and wild *G. pallidipes*, respectively, and 0% and 0–8.3% in laboratory and wild *G. p. gambiensis*, respectively. *Wolbachia* was not detectable in wild and laboratory populations of *G. p. palpalis*, *G. f. fuscipes* and *G. tachnicoides*.

Although the correlation between *Wolbachia* densities and the SGH incidence is yet to be experimentally demonstrated, data from various studies from laboratory-bred and field-collected *Glossina* spp., implicate a species-specific relationship between the occurrence of SGH and *Wolbachia* infections. For instance, whereas *Wolbachia* prevalence is high in various laboratory-bred colonies of *G. m. morsitans* and *G. m. centralis* (100%) and *G. brevipalpis* (41.2%) [70], there is no demonstrable evidence for the occurrence of overt SGH symptoms in these three *Glossina* species. However, this apparent inverse *Wolbachia*-*Glossina* relationship differs from some laboratory-bred *Glossina* species (e.g. *G. palpalis*, *G. p. gambiensis* and *G. f. fuscipes*) that have undetectable *Wolbachia* infections symptoms [70, 71] and so far, there is no evidence for the occurrence of overt SGH. It should be noted that despite the absence of diagnostic SGH symptoms, the above-mentioned species are susceptible to various degrees to GpSGHV infections, regardless of whether the infections are natural or artificial (intra-hemocoelic injections) [25, 27, 35]. On the other hand, laboratory-bred *G. pallidipes* flies, which typically exhibit overt SGH symptoms and high prevalence of GpSGHV (up to 100%) appear to be either *Wolbachia*-free [23, 72], or harbor extremely low densities of this symbiont [70, 71].

### Impact of symbionts on tsetse susceptibility to GpSGHV infections

Antibiotic-mediated suppression of the *Wigglesworthia*/*Sodalis* complex in *G. pallidipes* reduces vertical transmission of GpSGHV and inhibits development of overt SGH symptoms in F_1_ progeny [23]. Significantly, laboratory *G. pallidipes* flies do not have detectable *Wolbachia*, thus precluding verification of its effects on GpSGHV pathogenesis and transmission [23]. However, *Wolbachia* densities in the DENV-*A. aegypti*-*Wolbachia* system are extremely low in mosquito midguts, fat bodies and SGs, which correlates with a lack of resistance to DENV infections as compared to the DENV-resistant *A. aegypti* that harbors high *Wolbachia* densities [73]. The *Wolbachia*-mediated virus resistance in mosquitoes and *Drosophila* is observed in *Wolbachia*-transinfected hosts, i.e. species infected with native *Wolbachia* strains do not typically show a virus-resistant phenotype, but the native strains still confer their hosts with antiviral responses [60, 74–76]. The lack of immune-priming could be due to co-evolutionary trade-offs between the symbionts and the host’s immune system as well as other life-history traits [77]. It is therefore possible that the low *Wolbachia* densities in *G. pallidipes* increases its susceptibility to GpSGHV infections.

### Impacts of symbiont deficiency in housefly susceptibility to MdSGHV infections

Despite the absence (or titers that are too low to be detectable) of symbionts in the housefly, this insect passively harbors highly diverse non-symbiotic microbiota that are environmentally acquired and that vary significantly between individuals [49]. Compared to *Drosophila*, the housefly genome contains a substantial increase in the repertoire of genes involved in immune-related pathways [29], many of which are significantly upregulated during MdSGHV infections [16]. It therefore seems that the housefly has complemented its symbiont deficiency by evolving structural, biochemical, and behavioral mechanisms that serve as barriers to opportunistic pathogen infections. Additionally, non-symbiotic bacteria may manipulate oviposition behaviors of the house fly via volatiles produced by bacteria on conspecific eggs [78, 79]. This may impact the fly’s population dynamics, and thus indirectly impact MdSGHV infections. The symbiont-mediated reduction of pathogen proliferation in dipterans implies that the absence or presence of low densities of symbionts potentially influences the occurrence of only symptomatic MdSGHV infections and the high virus prevalence amongst housefly populations. Further, the housefly-associated microflora may directly correlate to the fly’s nutritional ecology and physiology [80], i.e. the housefly has significantly lower energy consumption compared to the relatively higher energy consumption associated with the specialized metabolism of tsetse flies [24].

## The ‘arms-race’ between SGHVs and their dipteran hosts

Evolution favors hosts that develop strategies to avoid or limit pathogen infections, and pathogens that develop effective mechanisms to evade the host’s anti-viral defenses. This ‘arms-race’ results in a stable but dynamic equilibrium (homeostasis) between a virus and its host [81], whereby the virus does not significantly compromise the host’s reproductive capacities, nor does the host’s immune system completely block production of virus progeny. In addition to the host immune pathways, non-immune responses such as cell-cycle control and signaling, are also involved in the establishment of the virus-host homeostasis at the cellular level [82]. Modulation of the non-immune pathways hinges on the dependency of viruses on the host’s machinery for gene expression [83], which can be regulated at the levels of transcription, protein turn-over, or transport of viral gene products and host’s transcription factors to viral replication sites within infected host cells.

### Host immune responses and SGHVs survival

The first line of insect’s defense is the physical barrier provided by the cuticle and the PM of the gut, in addition to, low pH, digestive enzymes and ROS it produces [84, 85] and various antimicrobial peptides (AMPs) that block the pathogen’s ingress/replication [86]. When breaching these defense barriers, pathogens elicit both cellular and humoral innate defenses within the insect body [84]. Known anti-viral responses in insects are largely elicited by the mere presence of virus-derived dsRNAs (RNAi-mediated degradation of viral genomes [87–89]), and by virus-induced cellular damages (via the Jak/STAT, Toll and Imd pathways [90–93]). Many of the genes in these pathways are significantly upregulated in SGHVs infections [16, 94–96].

Once established, the outcome of virus infection, whether acute or chronic, depends on the balance between the speed of viral replication/dissemination and the efficiency of the host’s immune responses. As a counter-defense, large dsDNA viruses possess many genes that can manipulate host immune responses [97]. These viruses have evolved to maximize their replication within the host by ‘acquiring and customizing’ strategic host genes to mimic, block and/or regulate key cellular processes, that confuse the host’s immune system and that enhance their dissemination. This ‘camouflage and sabotage’ paradigm is evolutionary advantageous to dsDNA viruses whose large and rather rigid genomes do not allow the high adaptive mutation rates associated with viruses containing smaller/single dsDNA or RNA genomes [98].

The GpSGHV virions incorporate several host-derived proteins, some of which potentially assist in evading the host’s immune system [95]. It is unclear whether MdSGHV virions incorporate host proteins. Unlike MdSGHV, GpSGHV appears to have recruited into its genome several cellular genes from its ancestral hosts during its evolution. The inheritance of host genes by the GpSGHV implies its long evolutionary relationship with the tsetse flies allowing the virus to construct a large genome that partially accounts for its dimorphic life style. On the other hand, the relatively smaller MdSGHV genome (120 kb) probably indicates that the housefly virus has not acquired as many genes (via horizontal gene transfer) as has GpSGHV (190 kb). Alternatively, MdSGHV may have had a longer evolutionary time with its host the housefly than GpSGHV with tsetse, in which case MdSGHV may have lost redundant genes. A third alternative explanation is that MdSGHV may have gained higher efficiency than GpSGHV in targeting beneficial host genes during infection so that a larger genome became evolutionary unnecessary.

## The host RNAi machinery and the SGHV’s evolutionary mechanisms

RNA interference (RNAi) is recognized as a conserved anti-viral defense mechanism in insects (and plants) that is not only active against RNA viruses but also against several groups of large dsDNA insect viruses (ascovirus, baculovirus, iridovirus and nudivirus) [99, 100]. RNAi involves short interfering RNAs (siRNAs; derived from exogenous dsRNA), and microRNAs (miRNAs; encoded by the host or viral genome), which interfere with gene expression by targeting specific mRNAs [101]. There is evidence for the presence and functionality of RNAi machinery against dsDNA viruses in flies, including the presence of the key genes in RNAi pathway (*Dicer-2* and *Argonaute-2*), and flies (e.g. *Drosophila*) with loss-of-function for these two genes are reported to be highly susceptible to viral infections [87, 88, 100, 102]. There are indications that both *Dcr-2* and *Ago-2* genes are not only expressed in tsetse flies, but are also significantly up-regulated in SGH symptomatic flies as compared with asymptomatic flies [103]. In the case of MdSGHV, at least two isoforms of *Ago-2* and *Dcr-1* genes were up-regulated in viremic females compared to their uninfected conspecifics [16]. Together, these data imply that the RNAi pathway may be actively involved in the dynamics of the SGHV-*Musca*/*Glossina* system.

The DNA virus-encoded RNAs are thought to act in a similar version to the host transcription factors and as such, they co-opt specific host gene pathways by destroying (e.g. in herpesvirus saimiri; HVS), boosting (e.g. in Epstein-Barr virus; EBV), or hijacking (e.g. in human cytomegalovirus; HCV) host miRNAs to reshape the cellular environment to the benefit of viral replication [104]. More importantly, due to their ability to attenuate the host immune responses, the virally-encoded miRNAs have been implicated in the reactivation of some viruses from latency [105]. During viral latency/persistence, only minimal numbers of genes are expressed to evade the host immune system [106, 107]. Examples of viral miRNAs involved in the latent/persistent virus infections include miR-H2-3p and miR-H6 (herpes simplex virus 1; HSV-1), miR-UL112-1 (HCMV), and miR-K5 (Kaposi’s sarcoma-associated herpesvirus; KSV) [108]. In some cases, certain viral miRNAs repress global expression of viral genes, for instance via epigenetic modifications of viral genomes [109, 110]. Significantly, it is more economical for viruses to evolve miRNAs targeting (complementary to) specific host mRNAs than it is to evolve novel regulatory genes. Besides, the small sizes of miRNAs (<200 nucleotides) do not pose size constraints on viral genomes.

In a genome-wide screen, Garcia-Maruniak et al. [13] identified six and seven miRNAs in the genomes of MdSGHV and GpSGHV, respectively. Although the presence of miRNA encoding sequences in the SGHVs were predicted in silico, it is likely that these miRNAs are functional. This is particularly so in the case of GpSGHV, which can switch from asymptomatic to symptomatic infections [2, 23], a transition that potentially depends on subtle differences in miRNA-mediated viral gene expression. In view of the subtlety of miRNA-mediated gene regulation, virus-encoded miRNAs may have reduced roles during MdSGHV symptomatic infection whereby robust viral and host gene expression may be dominant, despite the presence of miRNAs in infected cells. Our contention here is based on the currently known repertoire of viral miRNA (*n* > 200) [111], whose putative functions (e.g. prolonging lifespan of infected cells, regulating virus and/or host genes expression to limit symptomatic infections, etc.) suggest that most of these miRNAs may facilitate viral latency/persistence. In this case, GpSGHV is more likely than MdSGHV to utilize miRNAs. It is important to note that, the RNAi machinery merely minimizes, but does not eliminate viral infections. It is not clear why this is the case. However, adoption of alternative or additional layers of anti-viral mechanisms by insects may have made it unnecessary to invest in the optimization of RNAi and associated pathways. In summary, one could question the impacts of RNAi on MdSGHV infection in the housefly (exclusively symptomatic), as compared to GpSGHV infection in tsetse fly (predominantly asymptomatic).

## Evolution of host’s apoptosis and SGHVs escape strategies

In their hosts, viruses from diverse families induce apoptosis, a biochemically and genetically-regulated cell suicide process [112]. Apoptosis is an important pillar of the host’s innate immune response aimed to limit the time and host’s cellular machinery available for viral replication and dissemination [113]. Viruses cause apoptosis due to their inhibition of the synthesis of host proteins, which disrupts the balance between the synthesis and degradation of apoptosis inhibitors and activators/initiators. This imbalance results in the increase of the activators over the inhibitors thereby promoting apoptosis [114]. However, in some cases (e.g. influenza and sindbis virus; SINV), apoptosis can facilitate virus replication and dissemination [115]. In such cases, the virus-induced apoptotic response results in pinching off of virus-containing apoptotic bodies, which are subsequently phagocytosed by neighboring (uninfected) cells thus facilitating virus dissemination within the host without eliciting immune response [116].

Apoptosis is initiated and executed by *c*ysteine-dependent *as*partate-specific *p*rote*ases* (caspases) upon activation by apoptotic stimuli. Activated caspases consequently cleave their target substrates (e.g. protein kinases, signal transduction proteins, chromatin modifiers, DNA repair enzymes, inhibitory endonucleases, etc.) [117]. The model organism, *Drosophila*, encodes seven caspases (See Table 1 and the references thereof) [118, 119]. These include three apoptosis initiators - death regulator Nedd2-like caspase (DRONC/caspase-9), death-related ced-3/Nedd2 (DREDD/Caspase-8), and serine/Threonine-rich caspase-A (STRICA), in addition to four effectors - death associated molecule related to Mch2 (DAMM), *Drosophila* interleukin-1β-converting enzyme (DrICE), death executioner caspase-related to Apopain/Yama (DECAY/Caspase-3/7), and death caspase protein 1 (DCP-1) [120]. Several caspases have also been reported in mosquitoes (*Ae. aegypti* and *An. gambiae*), the red flour beetle (*Tribolium castaneum*), and the pea aphid (*Acyrthosiphon pisum*) [117]. Apoptosis in *Drosophila* is activated by DRONC, and dampened by *Drosophila i*nhibitor of *a*poptosis *p*rotein 1 (DIAP1). DIAP1 directly binds to two of the four effectors (i.e. DCP-1 and DrICE) to block their substrate-cleavage activities, and promotes ubiquitination of DRONC [119]. DREDD is essential for activation of the innate immune responses (via cleavage of Relish, a NF-κB family member of the Imd pathway); DREDD activity is dampened by Defense repressor-1 (Dnr1) protein [121]. The upregulation of DAMM upon DCV infections in *Drosophila* [91] suggests involvement of apoptosis in DCV infection, potentially via the JAK/STAT pathway.

**Table 1:** Apoptotic and/or immunity-related roles of *Drosophila* caspases

*Drosophila* caspase	GenBank Acc. No.	Apoptotic and, (or anti-viral roles in insects	Refs.
Apoptosis Initiators			
Death regulator Nedd2-like caspase (DRONC); Caspase-9 homolog	NP_524017.1	Ecdysone-induced (developmental and stress-induced apoptosis);	[108, 170]
Death-related ced-3/Nedd2 (DREDD) or DCP-2; Caspase-8 homolog	NP_477249.3	Essential for activation of innate immune signaling (activates Relish of the Imd pathway)	[108, 171]
Serine/Threonine-rich caspase-A (STRICA) or Downstream regulatory element-antagonist modulator (DREAM)	NP_610193.1	Together with DRONC, STRICA activates DCP-1 and DRICE	[172]
Apoptosis Effectors			
Death associated molecule related to Mch2 (DAMM)	AAF82437.1	Upregulated in DCV-infected *Drosophila*	[92]
Death caspase protein 1 (DCP-1)	NP_476974.1	Essential for germ-line apoptosis in mid-oogenesis; cleaves P35	[172]
Death executioner caspase-related to Apopain/Yama (DECAY); Caspase-3, (7 homolog	NP_477462.1	Involved in developmental apoptosis and immunity; (upregulated in DENV-infected mosquito)	[111, 171, 173]
Drosophila interleukin-1β-converting enzyme (DrICE)	NP_524551.2	Required for baculovirus-induced apoptosis; inhibited by the baculovirus P35/ P49/CrmA	[131]

**Fig. 1:**
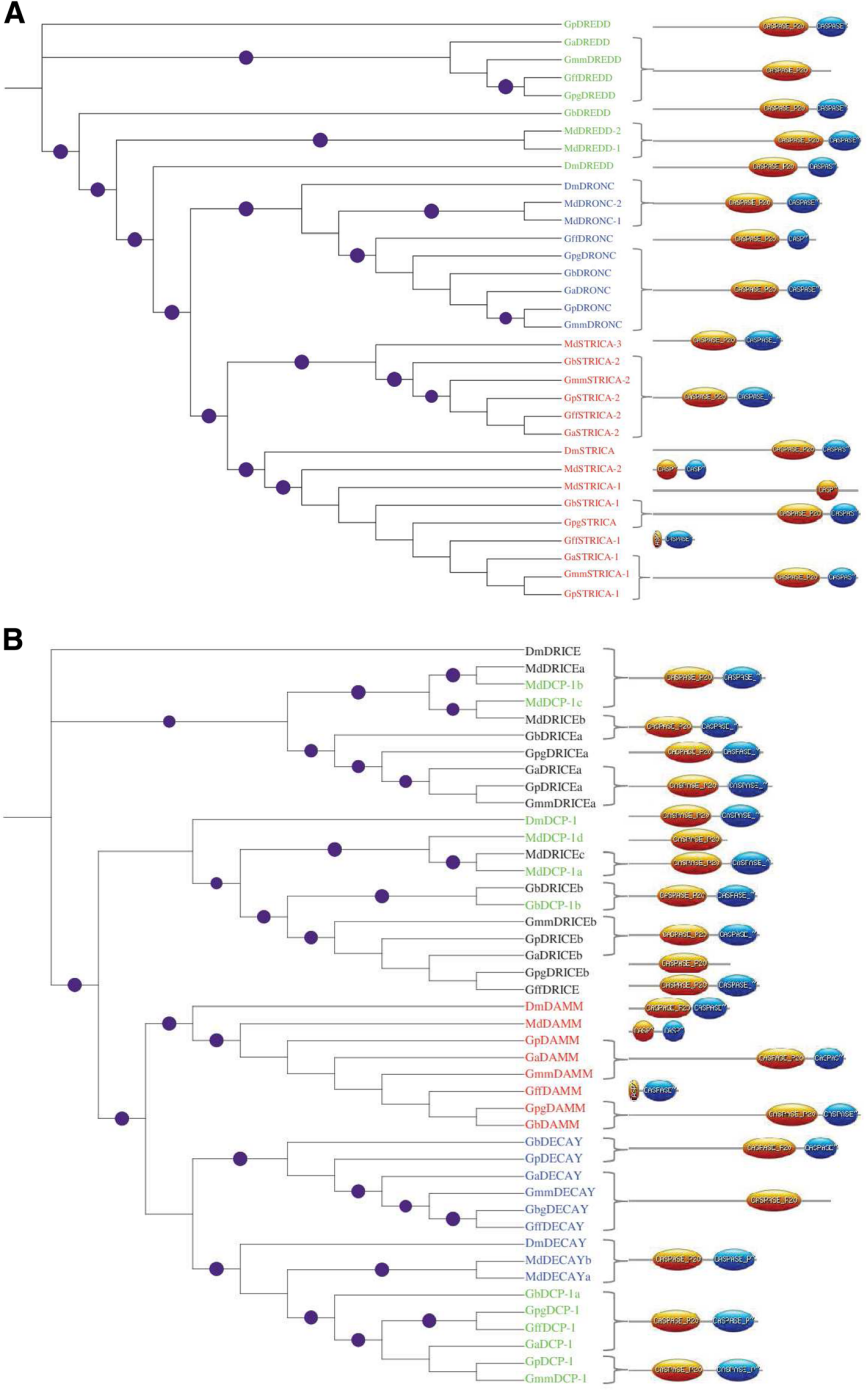
Phylogenetic analysis of initiator caspases (DRONC, DREDD and STRICA), and effector caspases (DAMM, DrICE, DECAY and DCP-1): **a** The three initiator caspases showed clear clustering across *Drosophila*, housefly and the *Glossina* species. **b** The effector caspase DAMM, and to a large extend DECAY, segregated clearly, but not for DRICE and DCP-1. Shown are the caspase prodomains of variable lengths, followed by p20 (orange) and p10 (blue) units that contain essential amino acid residues required for substrate recognition and catalysis. The prodomains were excluded during the phylogenetic reconstructions. Purple circles indicate bootstrap support of >80%

Using the seven *Drosophila* caspase sequences as baits, we searched various databases for *M. domestica* and *Glossina* spp., caspase homologs and performed phylogenetic analyses using the conserved caspase domains (Fig. 1). The three initiator caspases (DREDD, DRONC and STRICA) clustered into distinct clades in the three dipterans (Fig. 1a). *Glossina* species had single copies of DREDD and DRONC, but the two initiator caspases were duplicated in *M. domestica*. STRICA was evidently duplicated in both *Glossina* spp., and *M. domestica* pointing to putative adaptive evolution of this caspase. For the effector caspases, *Glossina* and *M. domestica* DAMM delineated into distinct orthologous clusters (Fig. 1b). *Glossina* DECAY caspases formed a distinct cluster closely related to the DCP-1 cluster into which *M. domestica* DECAY segregated. The *M. domestica* DECAY was apparently duplicated. All the *M. domestica* DCP-1 segregated together with DrICE caspases (Fig. 1b). Together, the analyses showed widespread duplication of *M. domestica* caspases as compared with their *Glossina* spp. homologs. The conservation of the caspases in *Glossina* and *M. domestica* imply functional conservation. We have recently reported that the caspase-8 (DREDD) homolog (essential for activation of innate immune response), caspase-3 (DECAY) homolog (apoptosis effectors), and Relish, were upregulated in viremic females compared to control houseflies [16].

To prolong infected cell viability and facilitate virus replication under the threat of apoptosis, viruses have evolutionary devised multiple mechanisms to inhibit apoptosis; these mechanisms mimic key regulators of apoptosis [122]. There are four main protein families of viral inhibitors of apoptosis, i.e. serpins, baculovirus P35 (and P49; P33), inhibitors of apoptosis (IAPs), and viral FLICE-inhibitory proteins. Three of these are well-known in large dsDNA insect viruses, i.e. P35 of the baculovirus *Autographa californica* MNPV (AcMNPV), its P49 homolog in *Cydia pomonella* granulovirus (CpGV), and the IAPs as present in *Orgia pseudotsugata* SNPV, CpGV [123] and many other baculoviruses. During infection in vertebrates, the baculovirus P33 (encoded by AcMNPV *orf92*) is involved in processes such as cell-cycle regulation, apoptosis, differentiations and oncogenic transformations [124]. P35-mediated inhibition of the evolutionary conserved ICE/ICE-like proteases [125] results in increased AcMNPV titers and allows successful virus infection in the host [126]. Neither MdSGHV nor GpSGHV encodes P35 or P49 homologs, which probably underscores the functional and evolutionary differences between SGHVs and baculoviruses. Further, SGHVs do not have homologs to baculovirus genes known to induce global protein synthesis shutdown, i.e. the host range factor-1 (HRF-1; *Lymantria dispar* MNPV), the host cell-specific factor-1 (HCF-1; AcMNPV), and the EP32 (*Hyphantria cunea* MNPV) [127]. However, MdSGHV encodes a single copy of IAP (MdSGHV078) [29] and *iap* transcripts are moderately enriched in viremic flies [16]. GpSGHV does not encode IAPs. The IAPs prevent apoptosis by acting at the evolutionary conserved signaling phase in the apoptotic pathway, i.e. block caspase activation [128]. IAPs prohibit apoptosis either via ubiquitination of host’s pro-apoptotic proteins (Grim, Reaper, HID and Sickle), or via direct interactions with caspases [129, 130], and may therefore act upstream of P35 or use the same targets.

**Fig. 2:**
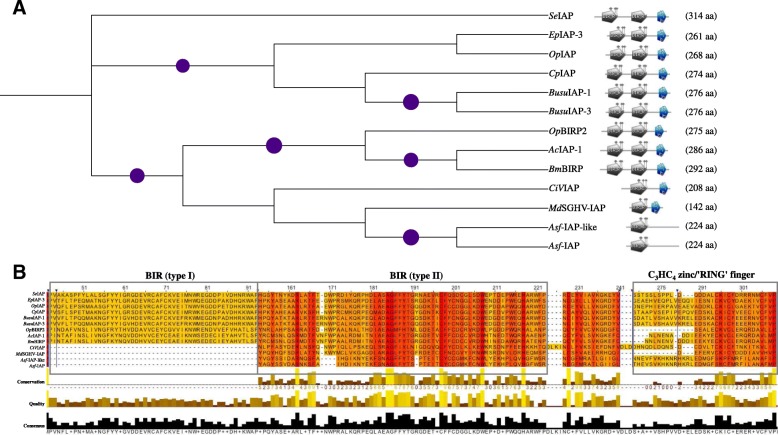
Phylogenetic analyses of IAP homologs from several viruses: **a** Phylogenetic clustering of MdSGHV IAP with homologs from *Spodoptera exigua* MNPV (seIAP), *Epiphyas postvittana* MNPV (EpIAP), *Orgia pseudotsugata* NPV (OpIAP/BIRP2, *Cydia pomonella* granulovirus (CpIAP), *Buzura suppressaria* NPV (BusuIAP-1/3), *Autographa californica* MNPV (AcIAP), *Bombyx mori* NPV (BmBIRP), *Chilo iridescent virus* (CiVIAP), and African swine fever virus (Asf-IAP/IAP-like). **b** Alignment of the IAP showing the functional baculovirus IAP repeat domains (BIR-1/2), and zinc binding fold. The IAPs from MdSGHV, AsfV and CIV contained a single BIR domain. Note that the irregular pentagons in Panel (**a**) represent types I and II BIR (in grey color), and the C_3_HC_4_ zinc/‘RING’ (Really Interesting New Gene) finger domain (in blue color), These regions are marked in Panel (**b**) of the figure (rectangular boxes)

We phylogenetically analyzed the MdSGHV IAP versus homologs reported in OpMNPV, *Spodoptera exigua* MNPV (SeMNPV), *Epiphyas postvittana* NPV (EppoNPV), and *Bombyx mori* NPV (BmNPV), *Buzura suppressaria* NPV (BusuNPV), CpGV, *Chilo iridescent virus* (CIV) and African swine fever virus (AsFV) (see Table 2). The MdSGHV IAP clustered with IAPs from AsFV and CIV (Fig. 2a). Compared to the domains of the IAPs from the other viruses, the MdSGHV, AsFV and CIV IAPs contained one BIR domain and an additional RING domain (Table 2; compare with Fig. 2b). Although cellular IAPs contain up to three tandem copies of the BIR domain, viruses contain one or two BIRs; a single BIR domain is sufficient for suppression of apoptosis [131]. The distinct domain organization within the IAPs potentially points to evolution by independent reductive changes from a larger ancestral sequence with complicated domain organization. It is also important to note that the housefly genome encodes for the pro-apoptotic proteins (Grim, Reaper, and HID) [29]. These data imply that during MdSGHV symptomatic infection, apoptosis does occur in the housefly, but that MdSGHV controls and alters progression of apoptosis to the benefit of the virus, i.e. to ensure that the infected cells do not only survive but also simultaneously grow and efficiently produce progeny virus. These events, combined with the multiple fronts employed by MdSGHV to manipulate host’s antiviral responses, may contribute to development of SGH symptoms.

**Table 2:** Comparison of MdSGHV IAP with homologs reported in other viruses: The analysis revealed clustering of the IAPs from MdSGHV, ASFV and CIV. Compared to the other viruses analyzed, the domain architecture of MdSGHV, ASFV and CIV IAPs contained one BIR domain and an additional RING domain (compare the data in this table with Fig. 2)

Virus	Sequence Name	GenBank Acc. No.	Length (aa)	BIR domain coordinates	RING domain coordinates
*Cydia pomonella* granulovirus (CpGV)	ORF17 iap-3	AIU36666.1	275	10–74; 111–176	224–269
*Orgyia pseudotsugata* multiple nucleopolyhedrovirus (OpMNPV)	IAP-3	NP_046191.1	268	21–85; 114–179	217–262
	IAP-1	NP_046197.1	275	27–92; 129–194	223–269
*Spodoptera exigua* multiple nucleopolyhedrovirus (SeMNPV)	IAP-3	CDG72862.1	314	34–99; 157–222	263–308
*Musca domestica* SGHV (MdSGHV)	IAP	YP_001883406.1	142	12–77	92–136
*Chilo/invertebrate iridescent* virus (CIV/IIV-6)	193R (BIRP)	NP_149656.1	208	40–109	159–203
*Autographa californica* nucleopolyhedrovirus (AcNPV)	IAP	NP_054056.1	286	32–97; 134–200	234–280
African swine fever virus (ASFV)	IAP	P0C9X4.1	224	32–93	94–224
	IAP-homolog	NP_042727.1	224	32–93	94–224
*Buzura suppressaria* nucleopolyhedrovirus (BusuNPV)	IAP-1	AAC34373.1	276	15–79; 111–177	225–270
	IAP-3	YP_009001870.1	276	15–79; 111–177	225–270
*Epiphyas postvittana* nucleopolyhedrovirus (EppoNPV)	IAP-3	NP_203195.1	261	14–78; 104–169	210–255
*Bombyx mori* nucleopolyhedrovirus (BmNPV)	IAP-1	NP_047432.1	292	32–97; 134–200	240–286

The apparent lack of anti-apoptotic gene homologs in GpSGHV suggests that this virus may follow alternative strategies to counteract their host’s apoptotic responses. An alternative anti-apoptotic strategy for viruses is to adopt a cryptic (asymptomatic) infection such that the productive virus infection is undetectable by the host’s immune surveillance, whereby only a subset of few viral genes is expressed. Perhaps this partially explains the asymptomatic infection state of GpSGHV, as well as the delayed development of SGH (i.e. SGH symptoms only develop in the F_1_ progenies produced by GpSGHV-injected mothers) [23]. Another strategy for a virus may be to adopt an immediate and rapid multiplication and assembly strategy soon after infection such that large amounts of its progeny virions are produced before the host mounts an effective apoptotic response. If the virus adopts this strategy, any apoptotic response from the host after virion assembly might enable dissemination of the virus within the host [108]. Significantly, in addition to possessing the *iap* gene, it is known that unlike GpSGHV, MdSGHV rapidly multiplies and induces detectable SGH symptoms in 100% of injected flies within 2–3 days post injection [3]. This rapid replication and possession of iap gene by MdSGHV may provide this virus with an additional layer of protecting itself from the housefly’s apoptotic response as compared to GpSGHV.

## Evolution of SGHVs and the host immune genes

### Immune-related genes in the SGHVs

As stated elsewhere in this review, the two SGHVs whose genomes have been fully sequenced have significant differences in their genome sizes and the number of genes, i.e. ~ 190 kb/160–174 genes and ~ 124 kb/108 genes in GpSGHV and MdSGHV, respectively. These differences raise the question of the evolutionary origins of the ‘novel’ genes in the GpSGHV-Eth genome in comparison to the genomes of the GpSGHV-Uga isolate and MdSGHV. To be noted is the fact that 37 MdSGHV genes are homologous to 42 genes in the GpSGHV-Uga genome (five MdSGHV genes are homologs to gene pairs in GpSGHV-Uga) [13]. Compared to the GpSGHV-Uga isolate, the GpSGHV-Eth isolate has added into its genome 24 putative ‘novel’ genes, but appears to have lost 11 genes and has 13 non-canonical genes (i.e. they have either CTG or TTG start codons instead of ATG) [6]. It is unclear whether the ‘novel’ ORFs in GpSGHV-Eth were due to criteria used to assign ORFs to the two GpSGHV isolates. However, four of the 24 ‘novel’ genes in GpSGHV-Eth harbor notable functional motifs. For instance the repetitive interspersed family (Rif/Stevor), viral small hydrophobic protein (v-SHP), and repeat-associated mysterious proteins (RAMPs) domains, which are important for gene duplication, virus docking and viral genome cleavage, respectively [6]. Moreover, another four of the ‘novel’ genes are both transcribed and translated, implying that they could be functional in GpSGHV-Eth [6]. Together, one could infer that these genes were co-evolutionarily acquired, followed by either their loss, and/or duplication of the essential domains to perform specific functions.

Nine of the MdSGHV/GpSGHV shared genes are homologs to the so-called core genes in baculoviruses [132], while 13 genes are unique to both SGHVs, but without homology to any known viral or cellular genes [13]. These shared SGHV genes can be considered as the ancient ‘core’ genes that have been vertically inherited from the ancestral or original member of the *Hytrosaviridae* family, and subsequently conserved amongst the descendants in this family. The remaining repertoire of ‘non-core’ genes, most of which encode proteins of unknown functions (PUFs) are presumably genus-specific (*Muscavirus* vs. *Glossinavirus*), or lineage-specific (GpSGHV-Eth vs. GpSGHV-Uga). The genus-specific genes may have been originally captured from the genomes of ancestral host species, followed by numerous duplication events. Similarly, the lineage-specific genes may have originated from unilateral gene transfers from unrelated viral (haplotype/isolate) genomes, followed by intra-genomic domain duplication and fusion events. Compared with the genome of the MdSGHV, the genomes of the two GpSGHV isolates harbor more ‘core’ genes found in the genomes of baculoviruses, nudiviruses, and entomopoxviruses (EPV) [132, 133]. These genes play critical roles in transcription of viral genes, viral packaging, assembly and egress. The core genes in the GpSGHV isolates that are not found in MdSGHV include Desmoplakin/Ac66 and core protein P4a (*Melanoplus sanguinipes* EPV), RNA polymerase transcription factor (*Amscata moorei* EPV), and the early/late gene expression factor-5 (LEF-5; *Culex nigripalpus* NPV) and LEF-8 (*Panaeus monodon* nudivirus; PmNV) [4, 6, 132]. Moreover, compared with the GpSGHV-Uga isolate, the genome of the more virulent GpSGHV-Eth isolate has distinct clusters of genes with deletions and insertions [6]. Together, these genomic differences between the SGHVs are pointing to a more complicated evolutionary history in the case of GpSGHV isolates compared to the MdSGHV.

Interestingly, unlike the core viral genes, most of the ‘acquired’ genes are not essential for virus replication, rather, these genes ensure a conducive cellular environment for virus replication [134, 135], for instance by mediating avoidance of the host’s immune system and prevention of inflammatory responses. Amongst these, genes encoding proteins that are homologs to host/cellular immunity-related proteins are particularly interesting because they are likely to have been acquired to mimic or interfere with the immune functions of their cellular homologs. This phenomenon is unique to DNA viruses because the RNA viruses utilize their high mutation rates to escape the host immune responses and persist in their hosts [97]. Examples are the virally-encoded interleukin-10 homologs by several DNA viruses, such as the homolog that potently suppress host immune responses against HCV [136], and the baculovirus-encoded IAP [137]. Notably, the homologies between the viral and cellular proteins may either be throughout the entire amino acid sequences, or only in conserved (functional) domains. Regardless of homology levels, these virally-encoded proteins outmaneuver their host-encoded homologs in modulating inflammatory and immune functions such that the virus is not eliminated and the host cell, in which case persistent infection state would prevail.

Table 3 details 14 of the GpSGHV-encoded proteins that are homologous to known cellular genes, of which only two have limited homologies (~ 20%) in MdSGHV, i.e. lecithine cholesterol acytransferase and glutathione S-transferase [4–6]. Nine of the 14 genes have been confirmed to be both transcribed (by RNASeq) and translated (mass spectrometry), implying that they are likely functional in GpSGHV infections [6]. Viruses can be viewed as an assembly of genes (i.e. their genomes are bits of different genes assembled together to form the genetic composition) [138], a phenomenon that partially accounts for the wide range of hosts from which the cellular genes originated (bacterial, parasites, nematodes, vertebrates and bacteriophages) (see Table 3). This is not unusual; some of the well characterized viral genes such as the DNA polymerases and helicases are of phage origin [139]. Nevertheless, the direction of horizontal gene transfers between viral and ancestral cellular genomes and/or vice versa is enigmatic. Whether the cell-derived genes mentioned in Table 3 have any roles in the evolution of GpSGHV is yet to be elucidated.

**Table 3:** SGHV-encoded orthologs of cellular genes: The protein families shown in this table were restricted to those that showed significant domain structural conservations. The proteins listed here have been described during the reporting of the genome sequences of the SGHVs [4–6]

Protein Name	GpSGHV ORF (ORF No.)		Location in virus particle	MdSGHV (ORF No.)	Homology or description
	GpSGHV-Eth	GpSGHV-Uga			
Lecithine-cholesterol acyltransferase ^(T, P)^	5	5	ICSVP ^£^	46	*Pseudomonas* sp.
D-3-phosphoglycerate dehydrogenase ^(T, P)^	6	7	Tegument	–	*Clostridium ultunense*
MAL7P1.132 ^(T, P)^	8	9	ICSVP	–	*Plasmodium falciparum* Str. 3D7
UDP-glucose-6 dehydrogenase ^(T)^	13	16	Unknown	–	*Pseudobutyrivibrio ruminis*
NADH ubiquinone oxidoreductase ^(T)^	30	29	Virion protein	–	Styphylococcal AgrD protein
Maltodextrin glycosyltransferase ^(T, P)^	39	38	Tegument	–	RGD-domain containing protein
Glutathione S-transferase ^(T, P)^	48	46	Tegument	84	Pre-mRNA splicing factor
Cellular protein CBG22662 ^(T, P)^	49	47	Tegument	–	*Coenorhabditis briggsae*
Rhoptry protein kinase ^(T)^	58	57	–	–	*Plasmodium yolei* Str. 17XNL
Signaling mucin HKR1	64	–	–	–	*Xenopus* (*Silurana*) *tropicalis*
RpoD protein ^(T)^	66	59	–	–	*Plasmodium falciparum*
ECF transporter ^(T, P)^	75	68	Envelop	–	–
Cellular protein PY00593 ^(T, P)^	124	113	Nucleocapsid	NaN	*Plasmodium yolei* Str. 17XNL
Tail length tape-measure ^(T, P)^	149	134	ICSVP	–	Oenococcus phage phi9805

Expression confirmed by transcriptomics (^T^) and proteomics only (^P^). Unmarked genes have no detectable transcripts or peptides; ^£^ These proteins do not have specific localization and were designated as ‘infected cell-specific viral proteins (ICSVP)’ [6]

### Immune genes in the housefly and tsetse fly

Virus evolution pressures their respective hosts to evolve immune counter-measures. Insect immunity consists of three main pillars. One pillar is the humoral immune response composed of the interconnected and synergistic Toll (immunity and developmental functions) and immune deficiency (IMD) pathways [140]. The second pillar consists of cellular responses (e.g. phagocytosis, PPO cascades) that result in pathogen phagocytosis and melanization [141], while as discussed above, the third pillar consists of the RNAi and other multipurpose pathways (e.g. JNK and the JAK/STAT).

The immune genes under these pillars can be broadly grouped in six functional categories, i.e. recognition, signaling, effectors, modulators, melanization, and RNAi (Table 4). The immune genes repertoires in *D. melanogaster*, *An. gambiae*, *G. pallidipes* and *M. domestica* show species-specific and extensive expansion of the recognition genes. For example, in *M. domestica*, calcium-dependent lectins (CTLs; *n* = 37) and thioester-containing proteins (TEPs; *n* = 22) have expanded when compared to *Drosophila* (34 CTLs; 10 TEPs), *G. pallidipes* (17 CTLs; 7 TEPs) and *An. gambiae* (25 CTLs; 13 TEPs) (see Table 4). The expansion of CTLs and TEPs in *M. domestica* implicates gene duplication driven by selective evolutionary pressures. The TEPs can be characterized as structurally unrelated opsonins that are critical for phagocytosis in many species, from insects to mammals [142]. The significant expansion of TEPs in *M. domestica* is probably an evolutionary necessity to enable this insect to deal with the plethora of diets/habitat-associated microbes. Compared to the other three insects, *M. domestica* seems to have acquired two additional homologs of the Down-syndrome adhesion molecule-1 (Dscam1), an insect opsonin equipped to cope with a broad range of pathogens [143].

**Table 4:** Major immunity genes in *M. domestica* and *G. pallidipes*: The immune genes described for the model insect, *Drosophila melanogaster* and the African malaria mosquito, *Anopheles gambiae* were obtained from the ImmunoDB [161]. The pathway for the putative immune-related proteins in *G. pallidipes* and *M. domestica* were verified by BLASTp searches at the Insect Innate Immunity Database (IIID) (≤10^−6^; bit score > 75) [164]. The pathways shown in this table have been reviewed by Kingsolver et al. [174]

Description of the functions and pathways of immune-related proteins in *Drosophila melanogaster*				Numbers of homologs		
Immune function	Key pathway	Protein name/sub-family	*D. melanogaster*	*An. gambiae*	*M. domestica*	*G. pallidipes*
Pathogen recognition	Lectin	Calcium-dependent (C-type) lectins (CTLs)	34	25	37	17
	Phagocytic	Down Syndrome cell adhesion molecule-1 (Dscam1)	1	1	3	1
		Pathogen pattern-recognition receptor Eater (Eater)	1	1	–	1
		Thioester-containing proteins (TEPs)	10	13	22	7
Signaling	Toll	Spätzle-like proteins (Spätzle)	6	6	4	7
		Toll receptors (Tolls)	9	10	7	6
		MyD88	1	1	1	1
		Tube	1	1	1	1
		Pelle	1	1	1	1
		TNF-receptor-associated factor-like (TRAF)	1	1	1	2
		Cactus	1	1	1	2
		Dorsal	2	–	1	8
Signaling	Imd	Immune deficiency (Imd)	1	1	1	–
		TGF-beta activated kinase 1 (Tak1)	1	1	1	1
		Kenny	1	1	–	1
		Inhibitor of nuclear factor kB kinase β (IKKb/ird5)	1	1	1	1
		Fas-associated death domain (FADD)	1	1	1	1
		Poor Imd response upon knock-in (PIRK)	1	–	1	–
		Caspar (Casp)	1	1	1	1
		TAK1-associated binding protein 2 (Tab2)	1	1	1	1
		Relish (Rel)	1	2	1	2
Signaling	JAK/STAT	Domeless	1	1	–	1
		Janus kinase (Hopscotch)	1	1	1	1
		Signal transducer and activator of transcription (Stat92E)	1	2	1	2
Signaling	JNK	Jun kinase (JNK)/basket	1	1	1	1
		Dual-specificity MAPK hemipterous (hep)	1	1	1	1
		Jun-related antigen (Jra/Jun)	1	1	–	2
Effectors	AMP	Antimicrobial peptides (AMPs)	21	11	21	4
		Lysozyme	17	8	29	5
		Nitric oxide synthase (NOS)	1	1	1	1
Modulators	Exocytic	CLIP-Domain Serine Proteases (CLIPs)	47	55	132	72
	Proteolytic	Serine protease inhibitors (serpins)	29	21	35	14
Melanization or Encapsulation	Humoral	Prophenoloxidase (PPO)	3	9	25	8
RNAi response	Small RNA Regulatory Pathways (SRRPs)	Argonaute (Ago)	3	3	2	3
		Armitage (Armi)	1	1	1	1
		Aubergine (Aub)	1	1	1	1
		Dicer (Dcr)	2	2	2	1
		Drosha	1	1	1	1
		Loquacious (Loqs)	1	1	2	1
		Partner of Drosha (Pasha)	1	1	–	1
		P-element induced wimpy testis (Piwi)	1	1	1	1
		R2D2	1	1	1	2
		Spindle-E (Spn-E) or Homeless	1	1	2	1
		Tudor staphylococcal nuclease (Tudor-SN)	1	1	1	1

The core immune signaling genes (Toll, Imd, JAK/STAT and JNK pathways) showed single-copy orthologs with similar divergence levels across the four dipterans (*D. melanogaster*, *An. gambiae*, *M. domestica* and *G. pallidipes*; see Table 4). Notably, although these pathways are traditionally thought to protect insects against infections by bacteria, fungi and parasites, evidence suggest that these pathways play a significant role in the defense against many viruses in both mosquitoes and *Drosophila* [83, 144]. Despite the overall conservation of the signaling immune genes in the four insects analyzed here, there are a few cases of gene losses and gains. For example, compared to the four Spätzle and one Dorsal protein homologs in *M. domestica*, *G. pallidipes* has expanded these two genes to seven *spätzle* and eight *dorsal* genes. Spätzle is an insect hemolymph cytokine, which in the moth, *Manduca sexta*, functions as a ligand that stimulates a broad-spectrum immune response to kill invading pathogens [145]. In *Drosophila*, Spätzle initiates a signaling cascade that culminates in the release of Dorsal from the protein Cactus to activate genes that are important for dorsal-ventral patterning in early embryonic development [145].

The effector and modulator gene categories seemed significantly diverged across the species, with the exception of single copies of nitric oxide synthase (NOS) in each species. There seems to be species-specific and extensive expansion of the modulator genes, with large expansion of the CLIP-domain serine proteases (CLIPs) and serpins in *M. domestica* compared to *G. pallidipes* (Table 4). In insect hemolymph, CLIPs proteolytically activate Spätzle (discussed in the previous paragraph) and other proteins [146], thereby serving as a potent mediator of insect immunity against invading pathogens. Most likely, the lifestyle, nutrition and ecology of the housefly have evolutionarily driven the selection of a large arsenal of effector and modulator genes to counter potential pathogens. In terms of the humoral responses, the expansion in the housefly of the prophenoloxidase (PPO) gene family (*n* = 25) is notable, especially compared to the significantly lower numbers of PPOs in *Drosophila* (*n* = 3), *An. gambiae* (*n* = 9), and *G. pallidipes* (*n* = 8) (see Table 4). In many arthropods, the PPO cascade is not only evolutionary conserved, but it is the primary extracellular pathway tasked with wound healing and melanization of infecting pathogens [147], which may be important for the ecology of the housefly. Further, the by-products of the PPO pathway have been reported to have antiviral effects in some viruses such as the baculoviruses [148–150], SINV [151], and Semliki Forest virus (SFV) [152]. The enrichment of the PPO pathway in *M. domestica* warrants further investigations of the extent to which the pathway is engaged in the pathogenesis of SGHVs.

**Fig. 3:**
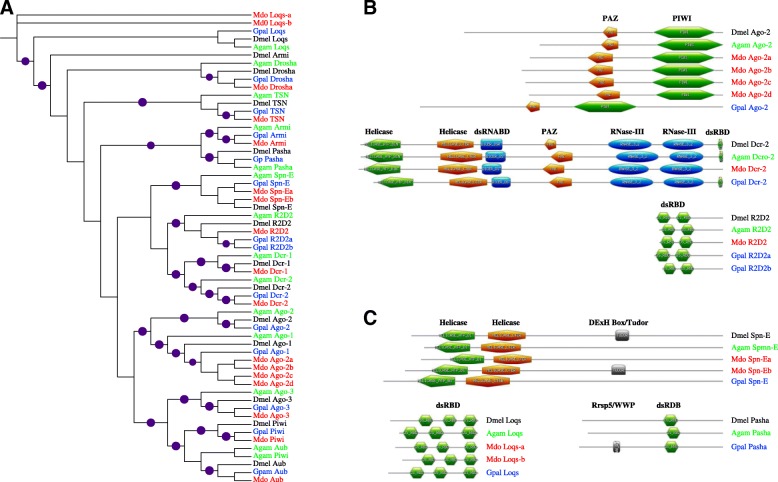
Phylogenetic analysis of the key RNAi pathway proteins in dipterans: **a** Clustering of the housefly and tsetse fly RNAi proteins with their homologs in the fruit fly and the malaria mosquito. **b** Domains of the three key RNAi pathway proteins, Ago-2, Dcr-2, R2D2, and **c** three of their main cofactors Spn-E, Pasha and Loqs. Purple circles indicate bootstrap support of >80%. The identities of the domains in the different irregular pentagon/hexagon shapes in Panels (**b**) and (**c**) in this figure are shown on top of each shape. Abbreviations: -PIWI, *P*-element *I*nduced *WI*mpy testis gene domain; PAY, PIWI-Argonaute-Zwille domains; dsRNABD/ dsRBD, double-stranded RNA-binding domain/motif; RNase-III, Ribonuclease III domain; DExH Box/Tudor, EAD/DEAH box helicases family protein domains; Rrsp5/WWP, rice root-specific promoter protein 5/Tryptophan-tryptophan-proline motif

The three key RNAi genes, *Ago-2*, *Dcr-2* and *R2D2* are arguably amongst the fastest evolving, and in *Drosophila*, they are subject to immense positive selection and selective sweeps [153, 154]. Compared to *Drosophila*, *An. gambiae* and *G. pallidipes*, *M. domestica* appears to have lost *Ago-1* and *Pasha*, but has acquired extra copies of *Loquacious* (*Loqs*) and *Spindle-E/Homeless* (*Spn-E/hls*) and expanded *Ago-2* (Table 4). On the other hand, *G. pallidipes* has duplicated *R2D2* (Table 4; Fig. 3). In *Drosophila*, Spn-E (and Piwi and Aubergine) is involved in the RNAi-mediated (via the piRNA branch) silencing of heterochromatin [155]. Specifically, *Spn-E* is required for activation of RNAi-mediated regulation of maternal mRNAs during oogenesis in *Drosophila* [156], and in defense against transposable elements [157], but its anti-viral roles are yet to be defined. One of the *M. domestica* Spn-E duplicates contained all the three signature domains found in the *Drosophila* Spn-E; the Spn-E homologs from mosquito and tsetse lacked the catalytic tetrad DExH box/Tudor domain (see Fig. 3c). However, absence of the catalytic tetrad is not unique because only a subset of the family possesses cleavage activity [158]. The three cofactors of Dcr and Drosha (Pasha, R2D*2* and Loqs) that are required in the first step of the RNAi pathway (i.e. generation of RNAs) [159, 160] contained the functional domains. Further, of the four AGO proteins (AGO 1–4), AGO-2 is singly capable of executing to the ultimate aim of the RNAi pathway (decimation of target mRNAs); knockdown of *Ago-1*, *-2* and *-3* genes does not compromise the integrity of the RNAi pathway [159]. This partially makes the loss of *Ago-1* gene in *M. domestica* inconsequential. Notably, the *M. domestica* Dcr-2 homolog lacks the dsRNA-binding domain (Fig. 3b), thus raising the question of what effects this has in the functionality of the protein.

The species-specific expansions and/or losses of the RNAi genes may have significant implications on the functionality of the pathway in tsetse and the housefly. Although it is yet to be experimentally validated whether all the RNAi key genes are expressed in the housefly, at least *Dcr-2* and *Ago-2* are expressed in *G. pallidipes*. Significantly, it has been proposed that RNAi is involved in the diversification and evolution of some viruses such as WNV and DENV in mosquitoes [144]. Consequently, given the complexity of GpSGHV haplotypes/isolates [6, 25, 27] compared to MdSGHV, one could surmise that the RNAi pathway might put a potent natural selection pressure on GpSGHV to favor evolution and maintenance of novel viral haplotypes.

## Conclusions

The data presented in this review suggest that the ecologies of the housefly and tsetse fly (i.e. how they interact with their environment or ecosystems either individually or as communities) and their life-history traits have influenced the persistence and transmission strategies, and hence the co-evolution between the host and the particular SGHV. In the case of the GpSGHV, so far, the absence of any known alternative reservoir host(s) may have necessitated the selection for vertical transmission (in a covert form) of the virus to facilitate its maintenance within tsetse fly populations between the SGH epizootics. Moreover, the existence of mixed modes of vertical and horizontal transmission may be evolutionary beneficial to the GpSGHV because it may contribute to the generation and maintenance of the virus diversity (haplotypes). For the MdSGHV, the possibility of existence of muscid hosts other than the housefly as reservoir or alternative hosts may have influenced selection for horizontal transmission of this virus. Additionally, the combination of horizontal transmission and the highly virulent nature of the MdSGHV (i.e. the virus infects only symptomatically) potentially hints to this virus as a regulating factor for housefly populations in a density-dependent or independent manner. However, further long-term studies are required to investigate the SGHVs infection and transmission dynamics and the roles of these viruses on regulations of the housefly and tsetse fly populations and community dynamics.

Many questions however remain unanswered with regard to SGHVs dynamics, particularly in natural populations of their respective hosts. For instance, why are GpSGHV-induced epizootics (SGH outbreaks) such rare occurrence in the field, and what are the genetic elements accounting for the differences in the pathogenesis of the two GpSGHV isolates? On the other hand, in the case of the MdSGHV for example, how is this virus maintained when the host populations fluctuate to low densities, and does the MdSGHV virulence (solely symptomatic infections) temporally and spatially modulate the community structures of the houseflies in nature? A further and even more challenging question is as to what extent the tripartite host-SGHV-microbiota interactions influences SGH epizootics. Finally, SGHVs are attractive ‘explorers’ to dissect the defense responses of their hosts and to study the transmission modes of large DNA viruses in dipteran flies.

## Methods

### Genome-wide identification of *G. pallidipes* and *M. domestica* immune genes

Annotated immune genes of *D. melanogaster* and *An. gambiae* sensu stricto were retrieved from ImmunoDB [161] and used to query (BLASTp; *e*-value ≤10^− 4^) the predicted proteomes of *G. pallidipes* Austen, and *M. domestica* Linnaeus (retrieved from VectorBase [162]). Canonical domains in the identified immune genes were ascertained using Pfam [163], and pathways confirmed by BLASTp searches in the Insect innate Immunity Database (IIID) [164].

### Phylogenetic analysis

To decipher phylogenetic relatedness of orthologous and paralogous immune genes in the genomes of *A. gambiae*, *D. melanogaster*, *G. pallidipes* and *M. domestica*, the retrieved sequences were aligned using MAFFT v7 [165], manually curated in Jalview v2.9 [166], and used for phylogenetic reconstructions using PhyML 3.0 [167] and MrBayes v3.2 [168]. The robustness of internal branches was evaluated using 100 bootstraps. The trees were rendered using iTOL v3.5.3 [169].

## Abbreviations

AcNPV: *Autographa californica* nucleopolyhedrovirus
Ago: Argonaute
AMP: Antimicrobial peptides
ASFV: African swine fever virus
BmNPV: *Bombyx mori* nucleopolyhedrovirus
BusuNPV: *Buzura suppressaria* nucleopolyhedrovirus
CA/CC: Corpora-allata/cardiaca glands
Caspase: Cysteine-dependent aspartate-specific protease
CIV/IIV-6: Chilo/invertebrate iridescent virus
CLIP: CLIP-domain Serine Protease
CpGV: *Cydia pomonella* granulovirus
CrPV: Cricket paralysis virus
CTL: C-type lectin
DAMM: Death associated molecule related to Mch2
DCP-1: Death caspase protein 1
Dcr: Dicer
DCV: *Drosophila* C virus
DECAY: Death executioner caspase-related to Apopain/Yama
DENV: Dengue virus
DIAP1: *Drosophila* inhibitor of apoptosis protein 1
Dnr1: Defense repressor-1
DREAM: Downstream regulatory element-antagonist modulator
DREDD: Death-related ced-3/Nedd2
DrICE: *Drosophila* interleukin-1β-converting enzyme
DRONC: Death regulator Nedd2-like caspase
Dscam1: Down Syndrome cell adhesion molecule-1
Eater: Pathogen pattern-recognition receptor Eater
EBV: Epstein-Barr virus
EppoNPV: *Epiphyas postvittana* nucleopolyhedrovirus
EPV: Entomopoxvirus
FHV: Flock house virus
GpSGHV: *Glossina pallidipes* salivary gland hypertrophy virus
HCV: Human cytomegalovirus
HRF-1: Host range factor-1
HSV-1: Herpes simplex virus 1
HVS: Herpesvirus saimiri
IAP: Inhibitor of apoptosis
IIID: Insect innate immunity database
Imd: Immune deficiency
KSV: Kaposi’s sarcoma-associated herpesvirus
LEF: Early/late gene expression factor
Loqs: Loquacious
MdSGHV: *Musca domestica* salivary gland hypertrophy virus
miRNA: MicroRNA
NOS: Nitric oxide synthase
OpMNPV: *Orgyia pseudotsugata* multiple nucleopolyhedrovirus
PPO: Prophenoloxidase
PUF: Protein of unknown function
RAMPs: Repeat-associated mysterious proteins
REL1: Relish protein
Rif/Stevor: Repetitive interspersed family
RNAi: RNA interference
ROS: Reactive oxygen species
RT-qPCR: Reverse transcriptase quantitative PCR
SeMNPV: *Spodoptera exigua* multiple nucleopolyhedrovirus
Serpin: Serine protease inhibitors
SFV: Semliki Forest virus
SINV: Sindbis virus
siRNA: short interfering RNA
SpexNPV: *Spodoptera exempta* nucleopolyhedrovirus
Spn-E/hls: Spindle-E/Homeless
Spz1A: Spätzle 1A
STRICA: Serine/Threonine-rich caspase-A
TEP: Thioester-containing protein
v-SHP: viral small hydrophobic protein
WNV: West Nile virus
